# Differences in the Volume of Pharmaceutical Advertisements between Print General Medical Journals

**DOI:** 10.1371/journal.pone.0084790

**Published:** 2014-01-08

**Authors:** Jennifer Gettings, Braden O'Neill, Dave A. Chokshi, James A. Colbert, Peter Gill, Gerald Lebovic, Joel Lexchin, Navindra Persaud

**Affiliations:** 1 Keenan Research Centre in the Li Ka Shing Knowledge Institute, St Michael's Hospital, Toronto, Ontario, Canada; 2 Department of Primary Care Health Sciences, University of Oxford, Oxford, United Kingdom; 3 Undergraduate Medical Education, Faculty of Medicine, University of Calgary, Calgary, Alberta, Canada; 4 United States Department of Veterans Affairs, Washington, District of Columbia, United States of America; 5 Division of Medical Communications, Brigham and Women's Hospital, Boston, Massachusetts, United States of America; 6 Department of Medicine, Newton-Wellesley Hospital, Boston, Massachusetts, United States of America; 7 Undergraduate Medical Education, Faculty of Medicine and Dentistry, University of Alberta, Edmonton, Alberta, Canada; 8 Applied Health Research Centre, Li Ka Shing Knowledge Institute, St. Michael's Hospital, Toronto, Ontario, Canada; 9 Assistant Professor, Institute for Health Policy Management and Evaluation, University of Toronto, Toronto, Ontario, Canada; 10 School of Health Policy and Management, York University, Toronto, Ontario, Canada; 11 Emergency Department, University Health Network, Toronto, Ontario, Canada; 12 Department of Family and Community Medicine, University of Toronto, Toronto, Ontario, Canada; 13 Department of Family and Community Medicine, St Michael's Hospital, Toronto, Ontario, Canada; Humboldt-Universität zu Berlin, Germany

## Abstract

**Background:**

Pharmaceutical advertisements have been argued to provide revenue that medical journals require but they are intended to alter prescribing behaviour and they are known to include low quality information. We determined whether a difference exists in the current level of pharmaceutical advertising in print general medical journals, and we estimated the revenue generated from print pharmaceutical advertising.

**Methods:**

Six print general medical journals in Canada, the United States, and the United Kingdom were sampled between 2007 and 2012. The number of advertisements and other journal content in selected issues of the Canadian Medical Association Journal (CMAJ), Canadian Family Physician (CFP), Journal of the American Medical Association (JAMA), New England Journal of Medicine (NEJM), British Medical Journal (BMJ), and Lancet were determined. Revenue gained from pharmaceutical advertising was estimated using each journal's 2013 advertising price list.

**Findings:**

The two Canadian journals sampled (CMAJ, CFP) contained five times more advertisements than the two American journals (JAMA, NEJM), and two British journals (BMJ, Lancet) (p<0.0001). The estimated annual revenue from pharmaceutical advertisements ranged from £0.025 million (for Lancet) to £3.8 million (for JAMA). The cost savings due to revenue from pharmaceutical advertising to each individual subscriber ranged from £0.02 (for Lancet) to £3.56 (for CFP) per issue.

**Conclusion:**

The volume of pharmaceutical advertisements differs between general medical journals, with the two Canadian journals sampled containing the most advertisements. International and temporal variations suggest that there is an opportunity for all general medical journals to reduce the number of pharmaceutical advertisements, explore other sources of revenue, and increase transparency regarding sources of revenue.

## Introduction

Print general medical journals remain an important medium for disseminating information to clinicians. The combined readership of six journals in the United Kingdom, the United States, and Canada is greater than 600 000[1]–[6] and, despite the importance of online content distribution, the number of clinicians reading print journals is growing. [7] Some medical journals display edited content such as peer reviewed research articles and practice pieces alongside advertisements.

The quality of information in print pharmaceutical advertisements is questionable. One independent review found that 86% of pharmaceutical advertisements contained accurate safety information about medications but that they also often contained misleading statements about efficacy (32%) that could lead to improper prescribing (44%), and most (57%) had little or no educational value. [8] Pharmaceutical advertisements have been criticized for making inaccurate or misleading claims, [9], [10] using poor quality references, [11], [12] and for failing to comply with government standards set to ensure appropriate use of the advertised products. [8], [13] There is no evidence that clinician exposure to pharmaceutical advertisements improves the process of care or clinical outcomes. [10]

A journal's source of revenue may be associated with its edited content. German medical journals that rely solely on revenue from pharmaceutical advertisements are more likely to include edited content that strongly recommends heavily promoted medications and patent protected medications than journals with no advertising revenue or mixed revenue sources. [14]

The revenue generated by pharmaceutical advertisements reduces subscription costs for subscribers and this has been cited as the reason journals continue to display ads. [15], [16] Differences in the volume of pharmaceutical advertisements and the revenue generated among journals would suggest the viability of other revenue sources (e.g., individual and institutional subscriptions, reprints, and other private advertising, including online advertising) for general medical journals. We compared the current (2007 – 2012) volume (number of pages) and concentration (ratio of pages of advertisements to journal content) of pharmaceutical advertisements in print general medical journals, determined whether current trends were also present in the past (1970 – 2012), and estimated the current revenue journals generate from pharmaceutical advertisements. Finally, we calculated the cost savings to individual subscribers that could be attributed to revenue from advertisements.

## Methods

To examine current differences between medical journals in Canada, the United States, and the United Kingdom, six journals were sampled between 2007 and 2012: *Canadian Medical Association Journal* (CMAJ), *Canadian Family Physician* (CFP), *Journal of the American Medical Association* (JAMA), *New England Journal of Medicine* (NEJM), *British Medical Journal* (BMJ), and *Lancet* (Table 1). These six journals were chosen because they are the general medical journals with the largest readerships in these countries, but they were not intended to be representative of all journals from the three countries. [1]–[6]CMAJ, JAMA, and BMJ were compared because they are published by the national medical association of each country. Three issues (the first issue in the months of February, May, and September) each year from 2007 to 2012 were sampled.

**Table 1:** General information regarding selected medical journals.

Journal	Frequency of Publication (issues per year)	Print Circulation	Impact Factor	Date of First Online Publication
**CMAJ**	18	69 404	6.5	
**CFP**	12	28 006	1.4	
**BMJ**	51	76 340	17	1995
**Lancet**	49	29 103	36	
**JAMA**	48	304 000	30	
**NEJM**	52	111 000	52	1996

The total number of pages in each sampled issue was recorded, and each page classified into one of nine categories:1) prescription medication advertisement; 2) non-prescription medication advertisement; 3) vaccine advertisement; 4) medical device/supply advertisement; 5) prescribing information (information about medications required by law in some countries, e.g., indications, side effects, contraindications, etc.); 6) other private advertisement; 7) government/NGO advertisements; 8) career/classified advertisement; and 9) journal content. Inserts were not counted. Advertisements for conferences or meetings were classified as career/classified advertisements. Advertisements for the journal itself (e.g., subscriptions, calls for papers, etc.) and prescribing/advertising indices were classified as journal content, along with edited content (e.g., research articles, cover pages, table of contents, etc.). In this paper, “pharmaceutical advertisements” refers to advertisements for prescription medications, non-prescription medications, vaccines, medical devices/supplies, and prescribing information, unless otherwise indicated.

Two reviewers initially abstracted data from three issues of CMAJ and CFP to verify agreement in the classification of the journal content. As NEJM, BMJ, and Lancet publish international editions of the journal, which may differ from domestic editions in the number of advertisements published, data from NEJM were abstracted in the United States, and data from BMJ and Lancet were abstracted in the United Kingdom. Three issues of JAMA were sampled by two reviewers (one in Canada and one in the United States) to verify that there were no differences in the advertising content of the international and domestic issues of JAMA. On this basis JAMA was sampled using the version found in Canada. Thus data from CMAJ, CFP, and JAMA were abstracted in Canada.

For the historical analysis, three issues (the first issue in the months of February, May, and September of the same year) in every even numbered year from 1970 to 2012 of CMAJ, CFP, BMJ and JAMA were sampled. Data from CMAJ, CFP, and JAMA were abstracted in Canada by one reviewer. Data from BMJ were abstracted by two reviewers in the United Kingdom.

Using the mean volume (i.e., total number of pages) of pharmaceutical advertisements and prescribing information in 2012, the amount of revenue from pharmaceutical advertising was estimated using rates from each journal's 2013 advertising price list or rate card. The print circulation was obtained from each journal's media kit and used to calculate the amount that individual subscribers would have to pay to replace the revenue that the journal gains from pharmaceutical advertising for 2012. All journals were contacted twice by email regarding the actual advertising revenue. Values are reported in British pounds using the average exchange rate for 2012 (1.5839 for Canadian dollars and 1.5853 for American dollars). [17], [18]

### Statistical Analysis

The volume of pharmaceutical advertisements was calculated for each sampled issue, as well as the ratio of pages of pharmaceutical advertisements to pages of journal content, hereafter termed the ratio of advertisements to content. A non-parametric analysis of variance (ANOVA) was used to determine whether there was a significant difference in the amount of pharmaceutical advertising in current (2007–2012) medical journals. For statistically significant results, non-parametric pairwise t-tests were used to compare volumes and ratios between association journals (CMAJ, BMJ, and JAMA). A p-value below 0.017 was considered significant after a Bonferroni correction factor of three was employed to correct for multiple comparisons.SAS version 9.3 (SAS Institute, Cary NC) was used for the analysis.

## Results

**Figure 1:**
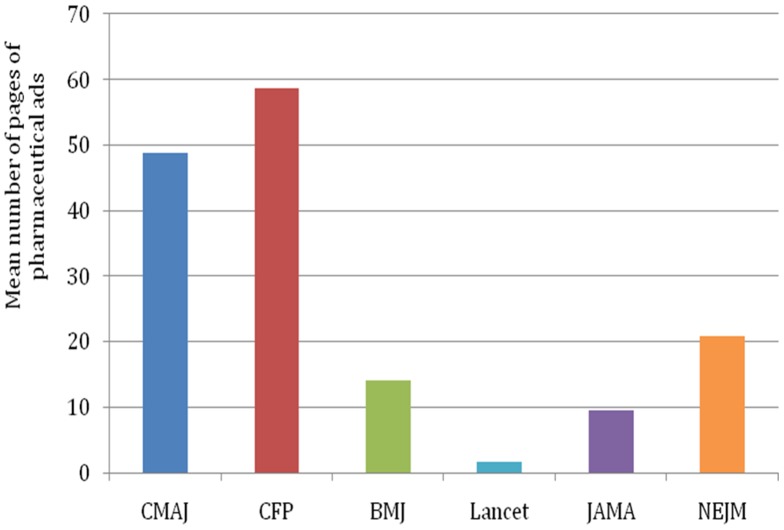
The mean number of pages of pharmaceutical ads, 2007 to 2012.

**Figure 2:**
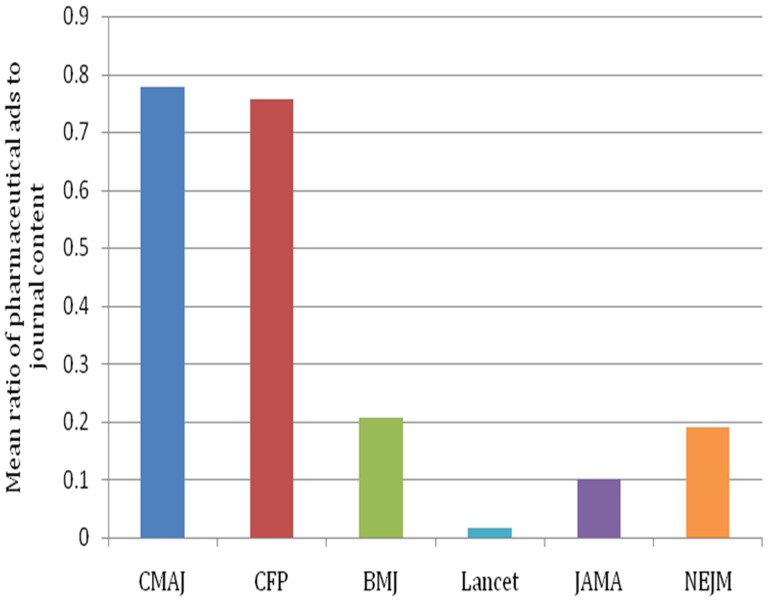
The mean ratio of the number of pages of pharmaceutical advertising to number of pages of journal content, 2007 to 2012.

Eighteen issues of CMAJ, CFP, JAMA, NEJM, BMJ, and Lancet were sampled between 2007 and 2012 for a total of 108 issues. The volume of pharmaceutical advertising in each issue ranged from 0 to 106 (0 to 54 excluding prescribing information) (Figure 1). The ratio of ads to content ranged from 0 to 1.7 (0 to 0.69 excluding prescribing information) (Figure 2).

There was a significant effect of journal on the volume of pharmaceutical advertising (chi-squared 88.16, 5 degrees of freedom, p<0.0001) and the ratio of ads to content (chi-squared 88.57, 5 degrees of freedom, p<0.0001). The volume of pharmaceutical ads in CMAJ was significantly greater than in JAMA (p<0.0001) and BMJ (p<0.0001). There was not a statistically significant difference between the volume of pharmaceutical ads in BMJ and JAMA (p = 0.043). The ratio of ads to content in CMAJ was significantly greater than in JAMA (p<0.0001) or BMJ (p<0.0001). The ratio of ads to content in JAMA was significantly greater than in BMJ (p = 0.0008).

There was a significant effect of country on the volume of pharmaceutical advertising in journals (chi-squared 41.1, 2 degrees of freedom, p<0.0001; Figure 1) and the ratio of ads to content (chi-squared 36.65, 2 degrees of freedom, p<0.0001; Figure 2). The volume of pharmaceutical ads was significantly greater in the two Canadian medical journals (CMAJ, CFP) compared to the two American medical journals (JAMA, NEJM) (p<0.0001) or the two British medical journals (BMJ, Lancet) (p<0.0001). The volume of pharmaceutical ads was significantly greater in the two American medical journals (JAMA, NEJM) than in the two British medical journals (BMJ, Lancet) (p = 0.0004). The ratio of ads to content was also significantly greater in the two Canadian medical journals (CMAJ, CFP) than in the two American medical journals (JAMA, NEJM) (p<0.0001) or the two British medical journals (BMJ, Lancet) (p<0.0001). There was no statistically significant difference in the ratio of advertisements to content between the British and American medical journals (p = 0.09).

**Figure 3:**
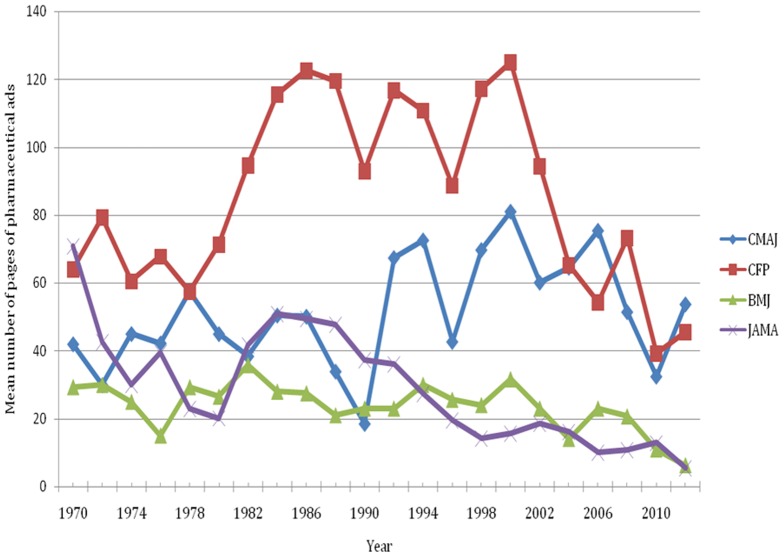
The mean number of pages of pharmaceutical ads (based on three issues per year, see text), 1970 to 2012.

**Figure 4:**
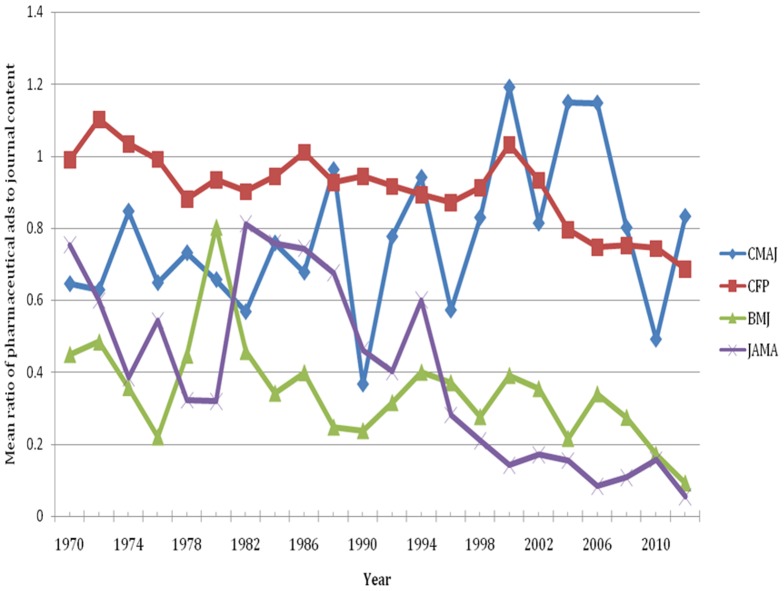
The mean ratio of number of pages of pharmaceutical ads to number of pages of journal content (based on three issues per year, see text), 1970 to 2012.

For the historical analysis, 65 issues of CMAJ, 66 issues of CFP, 37 issues of BMJ, and 66 issues of JAMA between 1970 and 2012 were sampled for a total of 234 issues. All data points for CFP and JAMA are the mean values obtained from three sampled issues in any given year. All data points for CMAJ are the mean of three sampled issues, except for 1986, which is the mean of the May and September issues. For BMJ: data points for 1970, 1978, 1982, and 2006–2012 are the mean of three issues; the data point for 1976 is the mean of two issues; the data points for 1972, 1974, 1980, 1984–2004 are the values from one issue. Other issues were unavailable for data abstraction after consulting three separate sources. The volume of pharmaceutical ads in each issue ranged from 0 to 149 pages (0 to 54 pages excluding prescribing information) (Figure 3). The ratio of ads to content ranged from 0 to 1.8 (0 to 0.69 excluding prescribing information) (Figure 4).

The difference between the Canadian journals sampled and the other journals is not a recent phenomenon (Figure 3). After 1990, the volume of pharmaceutical advertising remained the same or increased in the Canadian journals sampled, but steadily dropped in JAMA until 2012 (Figure 3). Some peaks and troughs in the volume of ads between 1986 and 2002 in CMAJ and CFP coincided. The volume of ads in CFP was quite variable between 1980 and 2012, whereas the ratio of ads to content was fairly constant (Figure 4).

### Ad Revenue

The estimated annual revenue from print pharmaceutical advertisements ranged from £0.025 million (for Lancet) to £3.8 million (for JAMA) (Table 2). The cost savings to each individual subscriber ranged from £0.02 (for Lancet) to £3.56 (for CFP) per issue. The CMAJ reported advertising revenue for 2012 as £1.6 million. The Lancet was unable to share the revenue figures due to confidentiality. The other journals did not respond to the request for information.

**Table 2:** Journal revenue from pharmaceutical advertising.

Journal	Mean volume of pharmaceutical ads in 2012 (number of pages)	Cost per page of advertising (2013), £	Mean volume of prescribing Information in 2012 (number of pages)	Cost per page of prescribing information (2013) £	Estimated Ad revenue per year (£ million)	Reported Ad revenue per year (£million)	Potential cost savings to individual subscribers per year, £	Potential cost savings to individual subscribers per issue, £
**CMAJ**	18.72	3 384	35.06	2203	2.5	1.6	23	1.26
**CFP**	26.04	2348	32.33	1193	1.2	-	43	3.56
**BMJ**	4.47	9 445	1.75	9 445	0.96	-	13	0.25
**Lancet**	0.25	1 534	0.08	1 534	0.025	-	0.86	0.02
**JAMA**	2.33	11 075	3.08	11 075	3.8	-	13	0.25
**NEJM**	4.00	8055	4.00	8055	3.3	-	30	0.58

## Discussion

There are significant differences between general medical journals in the volume of pharmaceutical advertisements. The Canadian general medical journals sampled contained over five times as many pages of pharmaceutical advertisements as the British and the American general medical journals sampled. The two Canadian journals had volumes of advertisements that were similar to JAMA before the 1990s when advertising in JAMA declined (Figure 3). Pharmaceutical advertising generates £0.025 to £3.8 million of annual revenue for each journal, saving individual subscribers between £0.02 to £3.56 per issue (all estimated values).

### Limitations

This study relied on the journals held in libraries and archives that are not always bound consistently. In some cases, the covers and some advertisements were not bound in the library copies of the journal, likely resulting in an underestimate in the number of ads since the inner front and back covers of journals often contain advertisements. Library copies do not generally include inserts. Three issues from each year rather than every issue of each journal were sampled even though journals publish different numbers of issues per year. We selected two journals from each country based on readership and while the medical association journals (CMAJ,BMJ, JAMA) may be comparable in many respects, the other sampled journals (CFP, Lancet and NEJM) differ with respect to target readership, scope and content. CFP is primarily read by Canadian family physicians while Lancet and NEJM reach an international audience of health professionals.

The revenue that journals gain from pharmaceutical advertising was estimated using each journal's rate for single full-page colour ads. Discounts are often provided to companies that pay for multiple ads. Multiple smaller ads on one page may account for more revenue than the amount paid for an individual full page. The estimated ad revenue included the revenue that journals gain from prescribing information. CFP has a special rate for prescribing information. Unlike the Canadian journals, American and British journals typically do not contain black and white pages of prescribing information that are separate from the pharmaceutical advertisement, thus the revenue from prescribing information was calculated using the cost of a full-page of colour advertising. Our estimate of advertisement revenue was higher than the reported revenue for the one journal that provided us information about journal revenue (CMAJ). The factors described above may account for the difference between the calculated and reported ad revenue for CMAJ. The discrepancy between the estimated and reported advertising revenue for the CMAJ suggests that the estimates for other journals may be higher than the actual value by as much as 40% and thus the effect of replacing revenue from advertisements with increases in subscription costs on individual subscriptions may be overestimated.

### Relationship with literature and Significance of findings

Some have argued that pharmaceutical advertisements have educational value and increase physician awareness of new drugs and therapies. [19], [20] There is no evidence that pharmaceutical advertisements improve the process of care or clinical outcomes [9] and claims made in advertisements can be misleading or inaccurate. [8], [21], [22] The information contained in some pharmaceutical advertisements does not support rational prescribing and has the potential to alter physician prescribing behaviour. [9], [10] In Canadian journals the advertisements are interspersed amongst the edited content of the journals while the prescribing information is found at the back of the journal where it may not be read.

Changes in the volume and ratio of advertising over time provide some insights into the evolution of journal business models and pharmaceutical industry marketing practices that may warrant future study. There was not a large change in advertising volumes when journals began publishing content online during the mid 1990s. The greater volume of advertising in Canadian journals compared with British and American journals dates back to the 1970s for CFP and the early 1990s for CMAJ. This suggests that the difference is not related to any recent developments such as changes in the publishing industry or general economic factors (e.g., the recession). The difference may reflect other changes in the journals' business models such as financial support from parent medical societies, changes in the size of the editorial staff, revenue from other sources (e.g., selling reprints, institutional subscriptions), the cost of journal production in different countries, and the number of copies of the journal printed (cost per issue decreases as the number of copies per issue increases). The covariation of the volume of ads in CMAJ and CFP in the historical analysis might indicate marketing campaigns that involved print advertisements in both journals in the same years. Indeed, we conducted a post-hoc analysis that showed certain products were advertised in both journals during peak but not trough years. The relative stability in the ratio of CFP ads to content and the volatility in the volume of CFP ads in the historical analysis suggest a causal relationship between the volume of edited content and the volume of ads in each issue. Perhaps either the number of pages of edited content determined the number of pages of advertisement or vice versa.

Differences in the volume of print advertisements between journals suggest that alternative sources of revenue, such as increased reprint prices and individual and institutional subscription costs, may be viable. For example, the cost of reprints for a single article published in Lancet generated revenue greater than £1.5 million [22] which exceeded print advertisements revenue generated in an entire year for CFP and BMJ. All revenue sources have potential negative consequences and this is certainly true of reprints. [16]

Based on the reported revenue from CMAJ, the revenue from pharmaceutical advertising potentially saves individual CMAJ subscribers approximately CAN $36 (£23) per year. If the revenue gained from pharmaceutical advertising were to be replaced completely by individual subscriptions, this would involve quadrupling the amount paid by members of the Canadian Medical Association for subscriptions to CMAJ (the annual Canadian Medical Association dues are CAN $450 (£284) plus provincial dues with CAN $12 (£8) allocated to CMAJ) but the total paid annually would still be one-fifth the non-member subscription rate (CAN $184 or £116) and a small fraction of mean physician income ($307 000 or £194 000).[23] The cost savings to individual subscribers for Canadian general medical journals is similar to that estimated for the Canadian Journal of Emergency Medicine.[24] Our results suggest that subscribers to American and British general medical journals potentially save less due to pharmaceutical advertising.

### Conclusions

The volume of pharmaceutical advertisements differs between general medical journals –with the Canadian journals sampled containing the most advertisements – and has decreased in the American and British journals sampled over time. International and temporal variations suggest that it may be possible for journals to limit pharmaceutical advertisements and explore other sources of revenue. In the interest of greater transparency, journals could report the amount of revenue received from the pharmaceutical industry (and other sources) from advertisements and reprint sales. This would help interested readers evaluate whether the integrity of the content is affected by the funding sources, just as readers are able to make such determinations for published papers that carry the author conflict of interest declarations mandated by journals.[25] Journals could disclose the number of advertisements and amount of advertising revenue generated in each issue and periodically (e.g., annually) report summary data to subscribers. Clinicians may prefer to avoid being exposed to pharmaceutical advertisements that are of questionable value by paying more for the edited content of general medical journals.
