# Correction: Differences in the Volume of Pharmaceutical Advertisements between Print General Medical Journals

**DOI:** 10.1371/journal.pone.0091628

**Published:** 2014-02-28

**Authors:** The PLOS ONE Staff

Information regarding a possible competing interest of the sixth author is incorrectly omitted from the Competing interests statement. The Competing interests statement should read: "James A. Colbert was an editorial fellow at the New England Journal of Medicine. Navindra Persaud is an Associate Editor for the Canadian Medical Association Journal. Peter Gill is a member of the Editorial Advisory Board of the CMAJ. All other authors have declared that no competing interest exists. This does not alter the authors' adherence to all the PLOS ONE policies on sharing data and materials."
